# Serious fungal infections in the Philippines

**DOI:** 10.1007/s10096-017-2918-7

**Published:** 2017-02-21

**Authors:** M. C. R. Batac, D. Denning

**Affiliations:** 1 University of Manchester, Manchester, UK; 2 Education and Research Center, University Hospital of South Manchester, Southmoor Road, Manchester, M23 9LT UK

## Abstract

The Philippines is a low middle-income, tropical country in Southeast Asia. Infectious diseases remain the main causes of morbidity, including tuberculosis. AIDS/HIV prevalence is still low at <1%, but is rapidly increasing. Fungal disease surveillance has not been done, and its burden has never been estimated. This becomes more important as the population of immunocompromised patients increases, drawn from patients with AIDS, TB, malignancies, and autoimmune diseases requiring chronic steroid use. Using the methodology of the LIFE program (www.LIFE-worldwide.org), estimates were derived from data gathered from WHO, UNAIDS, Philippine Health Statistics 2011, Philippine Dermatological Society Health Information System database, HIV/AIDS and ART registry of the Philippines, epidemiological studies such as The TREAT Asia HIV Observational Database 2005, and personal communication. Aspergillosis and candidiasis were the top causes of fungal infections in the Philippines. Chronic pulmonary aspergillosis (CPA), drawn from the number of tuberculosis patients, affects 77,172 people. Allergic bronchopulmonary aspergillosis (ABPA) and severe asthma with fungal sensitization (SAFS) frequencies, which were derived from the number of asthmatic patients, affect 121,113 and 159,869 respectively. Recurrent vulvovaginal candidiasis (RVVC) affects 1,481,899 women. Other estimates were cryptococcal meningitis 84, *Pneumocystis* pneumonia 391, oral candidiasis 3,467, esophageal candidiasis 1,522 (all in HIV-infected people), invasive aspergillosis (IA) 3,085, candidemia 1,968, candida peritonitis 246, mucormycosis 20, fungal keratitis 358, tinea capitis 846 and mycetoma 97 annually. A total of 1,852,137 (1.9% of population) are afflicted with a serious fungal infection. Epidemiological studies are needed to validate these estimates, facilitating appropriate medical care of patients and proper prioritization of limited resources.

## Introduction

In the Philippines, most of the initial epidemiological studies on mycoses focused on superficial fungal infections [1–3]. Since the 1950s, superficial mycoses have been in the top five most common diagnoses in outpatient dermatology clinics [2]. For several decades, fungal diagnostic procedures in the Philippines were not routinely done and were limited to the isolation, culture, and identification of fungi involved in skin, hair, and nail infections. Two university-based microbiology departments conduct short courses in Diagnostic Mycology on a regular basis. In the past, these used to cater solely for dermatologists, because the course was a requirement in their residency training. However, with the advent of AIDS and the compromised patient, more infectious disease fellows and medical laboratory personnel have enrolled in these courses.

The advent of AIDS, first described in the Philippines in 1984, led to a greater recognition of fatal fungal diseases [4]. Out of 470 HIV/AIDS patients seen in a tertiary general hospital in the Philippines, 32.5% presented with opportunistic infections [5]. Among the opportunistic infections, 32% were due to *Pneumocystis* pneumonia, 4% to cryptococcal meningitis, and 3% to oral thrush [5]. HIV cases are climbing steeply in the Philippines (20–30% annual increase in the years 2013–2016), especially among men [4]. Patients who are on immunosuppressive agents, or are immunocompromised due to medical conditions and/or are critically ill, are also at an increased risk of acquiring serious fungal infections, and such patients are common in the urban centres of the Philippines. There is no active surveillance done for serious fungal infections in the country, and epidemiological studies even for benign superficial mycoses have been sparse. Although nationally based surveillance programs are the gold-standard in estimating disease prevalence, they are usually costly and difficult to implement [6]. Therefore, the actuarial method used by previous researchers was used in this study to estimate the incidence and prevalence of serious fungal diseases, in which results from previous epidemiological studies, populations at risk, and epidemiological databases were used [6].

## Methods

Applying the methodology of the LIFE program (www.LIFE-worldwide.org), prevalence and incidence of serious fungal infections were derived from data gathered from WHO, UNAIDS, Philippine Health Statistics 2011, Philippine Dermatological Society (PDS) Health Information System database, HIV/AIDS and ART registry of the Philippines, epidemiological studies such as The TREAT Asia HIV Observational Database 2005, and personal communication.

## Results and discussion

### Population and country profile

The Philippines is classified as lower middle income by the World Bank. It is considered one of the promising newly industrialized countries, enjoying a steady economic growth at the turn of the millennium, with a per capita 2013 gross domestic product of $2,765. Based on the 2013 WHO statistical profile, the population of the Philippines is 98,394,000, with 34% under the age of 15, and 6% over the age of 60. The median age is 23. The number of hospital beds per population was the lowest in Asia in 2010 [7]. Table 1 shows relevant population statistics.

**Table 1:** Important population statistics

	Percent^a^	Number
Population (2013)		98,394,000
Males	50.5	49,688,970
Females	49.5	48,705,030
Below 15 years old	34.0	33,453,960
Above 40 years old	25.6	25,188,864
Above 60 years old	6.0	5,903,640
Females 15–50 years old	25.9	25,484,046

^a^(Epidemiology Bureau, Department of Health 2011)

### Burden of fungal infections

In 2016, a total of 1,852,137 severe fungal infections are estimated to have occurred in the Philippines. Table 2 shows the incidence and prevalence for selected serious fungal infections. Eighty percent of this is due to RVVC. However, local practicing obstetricians think this number is too high, and approximate that each general obstetrician sees 12–15 patients with vulvovaginal candidiasis, three to four of whom have RVVC per month. There are around 4,000 to 5,000 obstetricians in the Philippines, so the maximum estimate for the prevalence of RVVC would only be 240,000, ∼1.2 million less than the estimate. Those with RVVC who consult a physician do not represent everyone in the population, and many women suffer in silence. It is likely that many patients self-medicate, since patients may purchase topical antifungals without prescription or try local remedies. Clearly, this substantial issue needs addressing in the Philippines.

**Table 2:** Prevalence and incidence of selected serious fungal infections in the Philippines, 2016

Serious fungal iInfection	Estimation method	Totals		Rate/100,000
Cryptococcal meningitis	7% of new AIDS diagnosis	84	Annual incidence	0.09
Pneumocystis pneumonia	31% of new AIDS diagnosis	391	Annual incidence	0.40
Invasive aspergillosis (IA)	10% of AML + an equal number in other haematological conditions, 0.5% of renal transplant patients, 4% of liver transplant patients, and 1.3% of patients hospitalized for COPD	3,085	Annual incidence	3
Chronic pulmonary aspergillosis (CPA)	22% of cavitary pulmonary TB, 2% of non-cavitary pulmonary TB, annually, reduced by 15% for surgery and death.	77,172	Prevalence	78
Allergic bronchopulmonary aspergillosis (ABPA)	2.5% of adult asthmatics	121,113	Prevalence	123
Severe asthma with fungal sensitisation (SAFS)	33% of the most severe 10% of adult asthmatics	159,869	Prevalence	162
Candidaemia	2/100,000 general population, 1.5 in non-ICU and 0.5 in ICU	1,968	Annual incidence	2
Candida peritonitis	50% of incidence of candidemia in ICU	246	Annual incidence	0.25
Oral candidiasis	90% of HIV patients not on antiretrovirals and CD4 < 200 × 10^6^/L	3,467	Annual incidence	3.5
Oesophageal candidiasis	20% of HIV patients not on antiretrovirals and CD4 < 200 × 10^6^/l + 5% of HIV patients on antiretrovirals	1,522	Annual incidence	1.5
Recurrent vulvovaginal candidiasis (RVVC) (>4x/year)	5% of women 15–50 years of age	1,481,899	Prevalence	3,012
Mucormycosis	0.2 cases per 1,000,000 population	20	Annual incidence	0.02
Fungal keratitis	Based on cases seen at a tertiary government hospital in the NCR in 2015	358	Annual incidence	0.36
Tinea capitis	Based on cases seen at Philippine Dermatological Society training institutions in 2015	846	Annual incidence	0.86
Mycetoma	Based on cases seen at Philippine Dermatological Society training institutions in 2015	97	Annual incidence	0.10
Total serious fungal infection burden		1,852,137		

HIV/AIDS cases in the Philippines are few with a cumulative number of cases at 34,999 since the first case was reported in 1984. However, a sudden rise in cases has been observed since 2009, with 83% of cumulative cases diagnosed between 2009 to present. Most of the newly diagnosed cases are in the 25–29 age group, and among men who have sex with men [4, 8]. Our estimated incidences of *Pneumocystis* pneumonia, cryptococcal meningitis, and oral candidiasis were based on HIV/AIDS cases in the recent past, which were relatively low but are increasing rapidly. Adequate diagnosis of opportunistic fungal disease with the highly sensitive and specific cryptococccal antigen lateral flow device [9, 10] and *Pneumocystis* PCR are required [11, 12]. It is not known if disseminated histoplasmosis contributes to illness and death in AIDS in the Philippines, but it is likely. Histoplasmin reactivity has been documented, and at least nine cases of histoplasmosis have been reported [2, 13, 14]. Hand in hand with an effective AIDS prevention program and prompt and adequate treatment of cases with antiretroviral drugs, proper management of opportunistic fungal infections is required.

The Philippine prevalence of IA, CPA, ABPA, and SAFS, diseases caused by *Aspergillus* sp., has never been studied. However, several cases of pulmonary aspergillosis have been documented. In 1975, a case of pulmonary aspergillosis was reported in Manila in a 25-year-old male who presented with a 7-year history of hemoptysis without fever and anorexia, and who was diagnosed with tuberculosis but did not respond to anti-tuberculosis medicines [15]. From February 1982 to May 1990, 16 cases of pulmonary aspergillosis were seen at the Lung Center of the Philippines [16]. Nine of them had inactive pulmonary tuberculosis; 13 underwent lobectomy or segmentectomy or cyst removal. From Jan 2000 to December 2002, 37 patients were diagnosed with aspergilloma, and all were surgically managed. Ninety-two percent presented with hemoptysis and 95% had tuberculosis [17]. In a retrospective study done in a private hospital in Manila, there were 14 cases of aspergillosis seen from 1998-2004; five were diabetics and three had renal transplants. Eleven presented with pulmonary aspergillosis, and three had extrapulmonary aspergillosis [18]. A case of primary laryngeal aspergillosis in a 28-year-old who presented with fever, cough, and cold a day after giving birth by caesarean section was reported [19]. The Philippines is endemic for TB, and urban and rural areas have high rates of COPD [20]. TB and COPD are both associated with CPA, and therefore it is probable that there are many undiagnosed CPA cases, with some of them managed as resistant TB cases. It is important to determine baseline prevalence using available serological tests.

Candidemia among infants confined in the neonatal intensive care unit was reported to affect 56 of 7,830 infants in 2004, and 31 of 7,830 infants in 2005 [21]. A case–control study done in the same facility revealed that birthweight of 1,250 to 2,499 grams and gestational age of <28 weeks were significantly associated with candidemia (*p* < 0.05) [21]. The rate of candidemia in different countries varied from 1.2 to 25 per 100,000 population [22]. We have decided to use two per 100,000 population since exposure to risk factors for candidemia may be low, and there are remarkably few hospitals for this populous country [7]. In some developed countries, candidemia rates are increasing instead of decreasing and may be due to increased survival of patients with severe diseases or extremely low-birth weight, more aggressive interventions in the form of surgery, transplants, invasive procedures and devices, immunosuppressive therapy, and use of broad-spectrum antibiotics [22]. These are resources that are not accessible to most Filipinos.

In 1955, dermatologists surveyed the schools where two children with tinea capitis studied; however, no additional case was detected. Back then, experts surmised that tinea capitis was uncommon in the Philippines and was not a public health problem, and this seems the case to this day [2]. The common dermatophytes isolated were *Microsporum canis, Trichophyton tonsurans*, *T. mentagrophytes*, and *M. gypseum* [2]. A total of 9,180 (11.5% of new patients) consulted for superficial mycoses in ten PDS institutions in 2015. Fungal keratitis, tinea capitis, mycetoma, and RVVC are mycoses that affect young, productive, and otherwise healthy patients. These affect work productivity and quality of life. It is important to manage these diseases promptly to avoid complications.

The incidence of subcutaneous mycoses is rare in the Philippines, but these usually cause disfiguring lesions in young people, limiting their mobility and affecting quality of life [1]. By 1963, three patients with eumycetoma caused by *Penicillium funiculosum*, *Madurella grisea*, and *Scedosporium* (formerly *Monosporium*) *apiospermum* had been seen in the Philippines [23]. Eumycetoma of 20 years duration in a 40-year-old man was found to be caused by *Phialemonium* sp., and was treated with terbinafine 250 mg twice a day for 9 months followed by excision of redundant skin [24]. Another case was reported in 2009 of a 33-year-old man with draining sinuses and nodules on the left foot and thigh, from which *Madurella* sp. was isolated [25]. More recently, *Madurella mycetomatis* was cultured from the foot lesions of 9 years duration of a 33-year-old man [26]. Most of these patients were farmers. If eumycetoma presents with chronic nodules and draining sinuses, chromoblastomycosis appears as warty lesions. A few cases of chromoblastomycosis have also been reported in the Philippines, including a 58-year-old man who presented with verrucous lesions of 25 years and a 71-year-old man with warty lesions of 48 years duration [2, 27]. Both were caused by *Fonsecaea compacta*.

Challenges in the management of serious fungal infections in a resource-limited setting include timely diagnosis, proper antifungal intervention, patient compliance with long-term treatment, and high cost of antifungal treatment.
